# A Case Report of a Double Catastrophe: True Left Ventricular Aneurysm and Ventricular Septal Rupture Complicating Acute Myocardial Infarction and Presenting as Chronic Heart Failure

**DOI:** 10.7759/cureus.11292

**Published:** 2020-11-02

**Authors:** Suraj Khanal, Soumitra Ghosh, Anand K Mishra

**Affiliations:** 1 Cardiology, Post Graduate Institute of Medical Education and Research (PGIMER), Chandigarh, IND; 2 Cardiac Surgery, Post Graduate Institute of Medical Education and Research (PGIMER), Chandigarh, IND

**Keywords:** ventricular septal rupture, acute myocardial infarction, heart failure, ventricular wall aneurysm

## Abstract

A combination of left ventricular aneurysm (LVA) and ventricular septal rupture (VSR) in an acute myocardial infarction (AMI) patient presenting as heart failure is extremely rare. Here, we report a rare case of concurrent true inferoposterior LVA and VSR secondary to inferior wall myocardial infarction (IWMI) presenting as chronic heart failure (CHF). To the best of our knowledge, this is the third reported case in the literature.

A 61-year-old man, who had an IWMI about one month prior, presented with CHF. Echocardiography revealed true inferoposterior LVA and VSR. Coronary angiography revealed double vessel disease involving the right coronary artery (RCA) and left circumflex artery (LCX). Left ventricular angiography confirmed a large posterobasal aneurysm and VSR. The patient underwent successful coronary artery bypass grafting (CABG) and ventriculoplasty along with VSR patch repair.

## Introduction

Mechanical complications after acute myocardial infarction (AMI) are rare and account for <1% of the total cases. Left ventricular aneurysm (LVA) is the most common mechanical complication of ST-elevation myocardial infarction (STEMI). However, the incidence of LVA has decreased dramatically to 0.2% due to early revascularization therapy. The most common site of LVA is the anterior wall and true LVA involving the inferoposterior wall is rare, comprising only 9% of total cases [1-2].

Ventricular septal rupture (VSR) is another fatal mechanical complication of AMI. Post-infarction VSR is more common in anterior STEMI (60%) located in the anterior or apical portion of the interventricular septum (IVS) [3] as compared to inferoposterior STEMI (20-40%) located in the posterior or inferior portion of IVS [4]. However, the occurrence of LVA and VSR as two mechanical complications of AMI in the same patient is extremely rare. Most of the cases of STEMI with mechanical complications have an acute presentation and, if not treated urgently, can lead to cardiogenic shock with high mortality rates. There are few case reports on the late presentation of patients with mechanical complications. Here, we report an unusual case of concurrent true inferoposterior LVA and VSR after inferoposterior AMI, who presented late at our medical facility with symptoms of chronic heart failure (CHF).

## Case presentation

A 61-year-old male patient was admitted to our hospital with symptoms of CHF (New York Heart Association Class IV). He was diagnosed with inferior wall myocardial infarction (IWMI) about a month ago, did not underwent thrombolysis, and was on optimal medical therapy (OMT). The patient was a smoker, non-diabetic, non-hypertensive, and without any family history of coronary artery disease.

**Figure 1:**
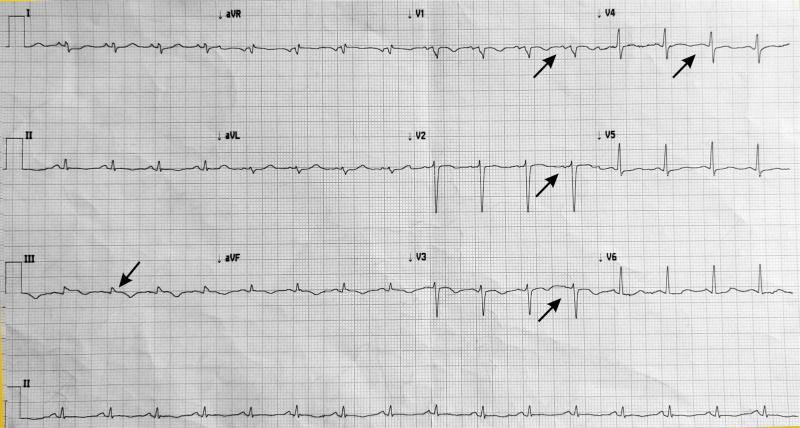
ECG showing sinus tachycardia, RAD, ST elevation in lead III, T inversion in lead II, III, aVF, and V1 to V4 ECG: electrocardiogram; RAD: right axis deviation

On examination, his vitals were stable along with edema and elevated jugular venous pressure. His cardiovascular examination revealed a holosystolic murmur (grade 4) at the left lower sternal border. Blood investigation revealed a normal level of creatinine kinase-MB (CKMB) and troponin I (cTnI) but elevated N-terminal-prob-type natriuretic peptide (NT pro-BNP). Figure 1 shows the findings of the electrocardiogram (ECG), that is, sinus tachycardia, right axis deviation (RAD), ST elevation in lead III, T inversion in lead II, III, aVF, and V1 to V4.

**Figure 2:**
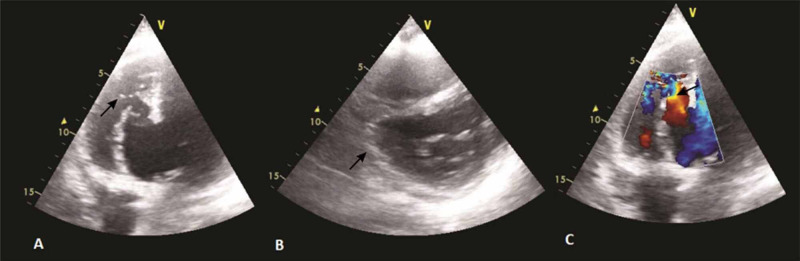
Echocardiography images Four-chamber view showing the aneurysm with ventricular septal rupture (VSR) (A); parasternal short-axis (PSAX) view showing aneurysm at right coronary artery (RCA) (B); Colour doppler showing flow across VSR (C)

Chest X-ray showed bilateral vascular congestion with mild bilateral effusion. Echocardiography (Figures 2a-2c) revealed a giant 41 x 40 mm posteriorly located submitral aneurysm opening into the left ventricle (LV) through 30 mm wide mouth and a small ventricular septal defect (VSD) with left to right shunt at the apex of the aneurysm with an area of 14 x 16 mm. Although the aneurysmal wall was akinetic, the remaining left ventricular segments were properly contacting, with left ventricular ejection fraction (LVEF) of 40%. The pulmonary systolic pressure estimated from the tricuspid regurgitation (TR) jet was 70 mmHg.

**Figure 3:**
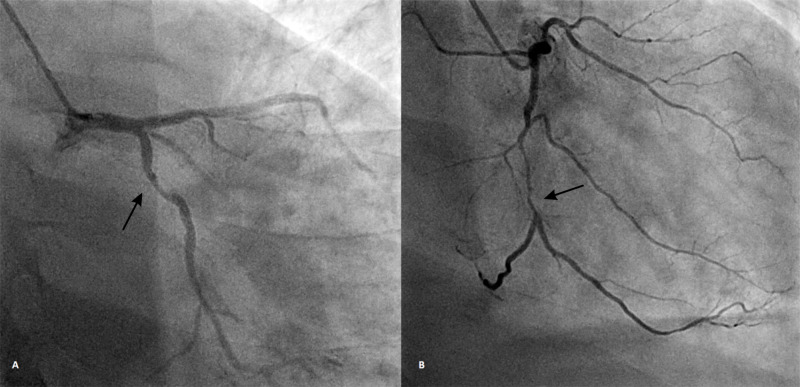
Coronary angiography AP-caudal view showing 80% occlusion of LCX (A); RAO view showing RCA (m) 90% occlusion of RCA (B) AP: anteroposterior; LCX: left circumflex artery; RAO: right anterior oblique; RCA: right coronary artery

The patient was admitted to the coronary care unit, and treatment was initiated with intravenous diuretics, dual antiplatelet therapy, statins, and unfractionated heparin. Coronary angiography (Figures 3a-3b) was done which revealed a proximal 80% occlusion of the left circumflex artery and mid 90% occlusion of the right coronary artery.

**Figure 4:**
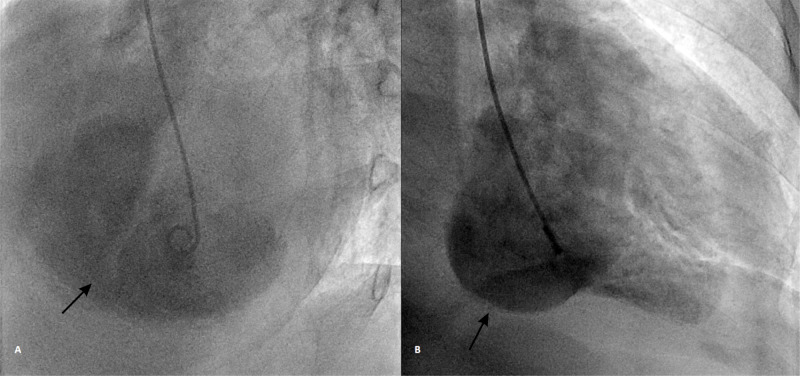
LV angiography. LAO Cranial view showing a VSD arising from the large LV aneurysm (A); RAO view showing large PB aneurysm (B) LV: left ventricular; LCX: left circumflex artery; LAO: left anterior oblique; VSD: ventricular septal defect; RAO: right anterior oblique; PB: posterobasal

**Figure 5:**
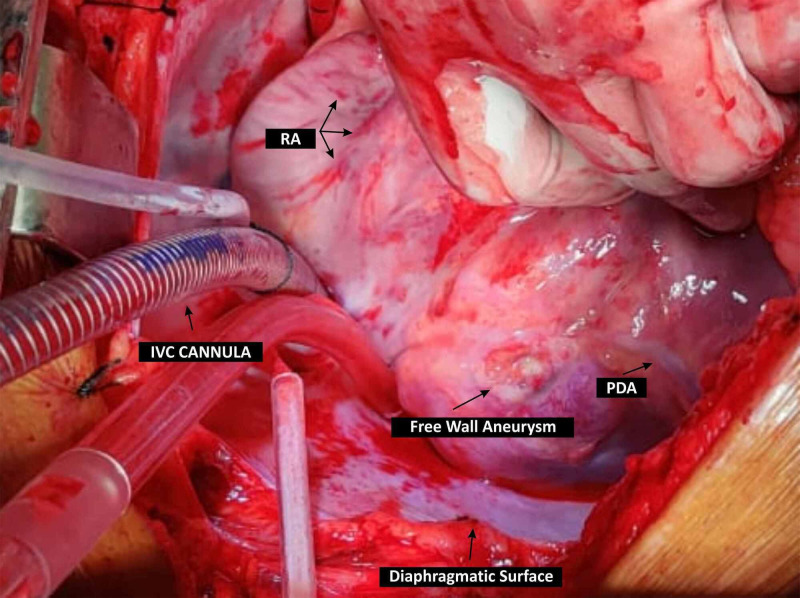
Intraoperative findings of the aneurysm RA: right atrium, IVC: inferior vena cava; PDA: posterior descending artery

Left ventricular angiography (Figures 4a-4b) was done that confirmed a large posterobasal aneurysm and VSD with a left to right shunt arising at the site of the aneurysm (Figure 5).

The patient underwent successful coronary artery bypass grafting (CABG) (saphenous vein graft to obtuse marginal and RCA) with left ventriculoplasty using a bovine epicardial patch of 45 x 40 mm with VSR repair. During surgery, there was a large true aneurysm of the posterior wall of LV near the base adjacent to distal RCA with a size of 45 x 40 mm along with a ventricular septal defect with a size of 15 x 15 mm on the LV side having an oblique course into the right ventricle (RV). The defect was present on the posterior IVS on the LV side with the opening in the RV just below the moderator band. The patient was shifted back after successful surgery on an intra-aortic balloon pump and extubated on the third postoperative day.

Echocardiography done after one week showed that the aneurysm was segregated from LV and there was no flow inside the segregated aneurysm. LVEF increased to 52% and pulmonary arterial systolic pressure estimated from the TR peak flow velocity dropped to 30 mmHg. The patient was discharged in stable condition on OMT.

## Discussion

LVA was first described by Fulton MN, based on the pathological finding of a dilated ventricle with a portion of the wall markedly thinned and replaced by fibrous tissue [5]. A true aneurysm is an abnormal protrusion of the ventricular wall and is typically formed two to three weeks post myocardial infarction (MI). The most common site of LVA is anterolateral near the apex (85%) and only 5%-10% of LVA involves the posterior wall [6]. Persisting ST-segment elevation, enlarged cardiac silhouette, and calcifications adjacent to the LV border on chest X-ray are suggestive of aneurysms. Eventually, this area develops hypokinesia and akinesia with an increased risk of thromboembolic events. Our patient had a posterior true wall aneurysm, which is quite rare. Continuous myocardium and laminar blood flow through a wide neck are the echocardiographic features of a true aneurysm [7] and all of these were present in our case.

VSR is a rare but fatal complication of AMI. Older age, female gender, prior stroke, and chronic kidney disease are the risk factors for VSR. In the current reperfusion era, VSR is rare and is generally diagnosed earlier during the post-infarction period. Mortality of VSR remains high (48%-87%) [8], even though the incidence has dropped to 0.17%-0.31% from 1%-2% [9]. Further, the timing of the diagnosis of VSR has decreased to 16-24 hours after AMI as compared to three to five days previously [10]. The location of VSR depends on the type of MI: generally anterior infarctions cause apical VSR and inferior or lateral infarctions cause basal VSR at the junction of the septum and the posterior wall. It causes left to right shunting of blood from LV to RV. The hemodynamic status of the patients depends upon several factors, such as the size of the defect, RV ischemia, RV infarction, or stunning of the RV from volume overload, and varies from complete hemodynamic stability to frank circulatory collapse. Doppler echocardiography has 100% sensitivity and specificity for the diagnosis of VSR [2]. Surgery is the treatment of choice in all patients even if clinically stable because the size of the septal defect can increase without warning. However, the timing of surgery is controversial. The American College of Cardiology/American Heart Association guidelines favor emergency surgery irrespective of the patient’s hemodynamic status [11]. On the contrary, the European Society of Cardiology guidelines favor delayed elective surgery in selected patients who respond to medical therapy [12]. The largest study of the society of thoracic surgeons database also showed that urgent surgery results in a high mortality rate (47-60%). In contrast, in patients who respond to aggressive medical therapy, delayed surgery has a better outcome, with operative mortality less than 15% [8]. The improved outcome with delayed surgery may be due to the evolution of the infarct, which allows more effective surgical repair. Our patient also had delayed surgical repair, although unintentional, and had a good clinical outcome.

Mortality with an IWMI, when complicated with VSR, is substantially high. But our case is exceptional, as the patient was hemodynamically stable with the symptoms of gradually progressive heart failure for almost one month. It might be because a giant aneurysm may act as a buffer area to shunting flow thereby preventing the surge of pulmonary artery pressure.

There are only a few case reports in the literature for concurrent true vertical reduction aortoplasty and VSR after MI [13-14]. While the first case was a 65-year-old female, the second case was a 49-year-old male patient. Similar to our case, in both cases, the patient had a good clinical outcome after surgery. Our case is rare and unique in many ways. First, aneurysms in the inferoposterior wall are uncommon. Second, VSR after MI rarely occurs in the posterior or inferior portion of the interventricular septum. Third, concurrent true aneurysm and VSR is very rare. Fourth, our patient presented a rare case of VSR with symptoms of CHF. Our case is probably the third reported case on the subject matter and demonstrates the clinical utility of CABG and ventriculoplasty along with VSR patch repair for a favorable outcome despite late presentation.

## Conclusions

Mechanical complications after AMI are rare but still prevalent. Concurrent true inferoposterior LVA and VSR secondary to IWMI can present as symptoms of CHF. High clinical suspicion and thorough physical examination at first medical contact can lead to the early identification of these cases. Echocardiography and LV angiography are valuable in diagnosing these types of rare cases. Combined CABG and ventriculoplasty with VSR patch repair are effective in treating this often fatal complication of inferior STEMI.
